# Exsection of the Sciatic Nerve for Neuralgia of the Stump Following Amputation of the Thigh

**Published:** 1859-03

**Authors:** Geo. C. Blackman

**Affiliations:** Professor of Surgery in the Medical College of Ohio; Surgeon to the Commercial Hospital


ART. III.—*Exsection of the Sciatic Nerve for Neuralgia of the Stump following Amputation of the Thigh.* By GEO. C. BLACKMAN, M.D., Professor of Surgery in the Medical College of Ohio; Surgeon to the Commercial Hospital.

ON the 7th of April last I received, from a physician of Preble County, a letter detailing briefly the sufferings of a sister who had been subjected to three amputations. From the above letter I make the following extract:—

"She has already had three amputations performed, and still suffers as much, if not more than ever. She first began to suffer in the year 1847. Thinks the pain was brought on by cold. Before the first amputation it felt as if suppuration was about to take place; was swollen to some extent, and was constantly cold from the knee to the foot. It was amputated about three years after the commencement of her sufferings. The synovial membrane and articular cartilage were found diseased. No benefit was derived from the first operation, which was performed just above the knee.

"The second amputation was done five months after the first. The pain did not cease for about a year, after which there was no return for five years. Third amputation was performed in October, 1856, and no relief followed. The pain is now (April 5th) the same as before the first amputation. It is not equally severe at all times. It is seated in the end of the stump, which is very painful when touched. She describes it as an aching pain; a sensation of drawing as if something were pressing tightly over the end of the bone. She has tried all, or nearly all, remedies in the Materia Medica, and none have given even temporary relief."

I visited this patient on the 15th of April, and found a lady about thirty-five years of age, with a countenance indicative of protracted suffering. Her menses were regular, and notwithstanding her great suffering she was but little emaciated. In view of the obstinacy of the case, after the faithful trial of numerous remedies, I concluded to try exsection of the great sciatic nerve. Assisted by Dr. Sloat, of Morning Sun, Ohio, and Drs. Red and Goodrich, of Oxford, I made my incision so as to expose the nerve as it passes between the great trochanter of the femur and the tuberosity of the ischium. I then removed an inch and a half of the trunk of the great sciatic, and as the small sciatic nerve passed directly over it, a portion of the latter was also included. The patient soon rallied from the effects of the chloroform, and could bear any amount of pressure on the end of the stump, when, before the operation, the contact even of the bed-covering, would throw the limb into spasm and produce the most excruciating pain. On the 19th of April her brother wrote me as follows: "My sister is still doing well. Appetite slowly improving. Rests very well at night; has passed two nights without morphia. There is no pain at the end of the stump, and no drawing sensation; but complains of some pain higher, in the vicinity of the wound."

A plaster of belladonna and opium was applied to the spine, and quinine and iron administered internally. The wound was slow in healing, and for five weeks she was confined to her bed. On the 19th of May she told the family physician, Dr. Sloan, that she was more free from pain than she had been for eight years. On the 1st of August I received a visit from her brother, from whom I learned that my patient was able to walk or ride, and that although not perfectly cured, her condition was far more comfortable than it had been for years. Her general health was excellent.

